# Cardiopulmonary Effects and Pharmacokinetics of Dexmedetomidine Used as an Adjunctive Analgesic to Regional Anesthesia of the Oral Cavity with Levobupivacaine in Dogs

**DOI:** 10.3390/ani12091217

**Published:** 2022-05-09

**Authors:** Matic Pavlica, Mojca Kržan, Ana Nemec, Tina Kosjek, Anže Baš, Alenka Seliškar

**Affiliations:** 1 Small Animal Clinic, Veterinary Faculty, University of Ljubljana, 1000 Ljubljana, Slovenia; matic.pavlica@vf.uni-lj.si (M.P.); ana.nemec@vf.uni-lj.si (A.N.); 2 Department of Pharmacology and Experimental Toxicology, Faculty of Medicine, University of Ljubljana, 1000 Ljubljana, Slovenia; mojca.limpel@mf.uni-lj.si; 3 Department of Environmental Sciences, Jožef Stefan Institute, 1000 Ljubljana, Slovenia; tina.kosjek@ijs.si; 4 Jožef Stefan International Postgraduate School, 1000 Ljubljana, Slovenia; 5 Faculty of Education, University of Ljubljana, 1000 Ljubljana, Slovenia; annze.bas@gmail.com

**Keywords:** regional anesthesia, nerve blocks, local anesthetics, adjuvant, dog

## Abstract

**Simple Summary:**

The modern approach to multimodal analgesia is based on the use of various types of analgesics before, during, and after surgery, including local anesthetics, such as levobupivacaine. Dexmedetomidine is used as an adjunctive analgesic to enhance the efficacy and duration of local anesthetics, but its use may be associated with dose-related adverse effects, such as hypertension and bradycardia. The primary aim of the study was to investigate the cardiopulmonary effects of dexmedetomidine administered perineurally as an adjunctive analgesic to regional anesthesia of the upper and lower jaw with levobupivacaine in anesthetized dogs. The secondary aim of the study was to investigate the pharmacokinetics of dexmedetomidine to explain the changes in cardiopulmonary parameters due to dexmedetomidine administration. Infraorbital administration was associated with faster absorption of dexmedetomidine, while application into the submucosa near the inferior alveolar nerve resulted in better bioavailability and faster elimination. Perineural administration of dexmedetomidine was associated with lower plasma concentration compared to intravenous administration and may be preferable because it induces a less pronounced cardiovascular response in terms of hypertension and bradycardia.

**Abstract:**

This study investigated the cardiopulmonary effects and pharmacokinetics of dexmedetomidine (DEX) used as an adjunctive analgesic for regional anesthesia of the oral cavity with levobupivacaine in anesthetized dogs. Forty dogs were randomly assigned to four groups of 10 dogs. All dogs received levobupivacaine (4 blocks) with DEX IO (infraorbital block, *n* = 10) or IA (inferior alveolar block, *n* = 10) or placebo (PLC; *n* = 10) or DEX (*n* = 10) was injected intravenously (IV) after administration of levobupivacaine. The dose of DEX was always 0.5 µg/kg. Cardiopulmonary parameters were recorded, and blood was drawn for the quantification of DEX in plasma using LC-MS/MS. Heart rate was lower in all LB + DEX groups, while mean arterial pressure (MAP) was higher in the LB + DEX IV and LB + DEX IA groups compared to the LB + PLC IV group. Compared to DEX IV, IO and IA administration resulted in lower MAP up to 2 min after application. Absorption of DEX was faster at IO administration (C_max_ and T_max_ were 0.47 ± 0.08 ng/mL and 7.22 ± 1.28 min and 0.76 ± 0.09 ng/mL and 7.50 ± 1.63 min for the IO and IA block, respectively). The IA administration resulted in better bioavailability and faster elimination (t_1/2_ was 63.44 ± 24.15 min and 23.78 ± 3.78 min for the IO and IA block, respectively). Perineural administration of DEX may be preferable because of the less pronounced cardiovascular response compared to IV administration.

## 1. Introduction

Modern anesthetic protocols are based on a multimodal approach to pain prevention that includes the use of various types of analgesics before, during, and after surgery. Perineural administration of local anesthetics (LA) causes efficient and reversible inhibition of excitatory conduction through the nerve and blocks the transmission of pain to the central nervous system. In dogs and cats, the anesthesia-sparing effect of regional anesthesia limits the adverse effects of volatile and injectable anesthetics, prevents hyperalgesia, reduces the consumption of analgesics during the postoperative period, and facilitates faster recovery from anesthesia [1,2,3,4]. 

Maxillary, infraorbital, major palatinal, inferior alveolar, and mental blocks are used to desensitize the maxilla and mandible in dogs undergoing invasive dental procedures [5]. Perineural administration of LA into the infraorbital canal in dogs desensitizes the soft and hard tissues from the first incisor to the first molar of the ipsilateral side of the maxilla. Desensitization of the soft and hard tissues of the ipsilateral side of the mandible is achieved by perineural administration of LA into the submucosa at the opening of the mandibular foramen where the inferior alveolar nerve enters the mandibular canal [3].

The most used LAs in veterinary medicine are lidocaine, bupivacaine, and ropivacaine [6,7,8]. After perineural administration of lidocaine into the infraorbital canal, desensitization of the area occurs after 2 to 5 min and lasts for 2 to 3 h, while after administration of bupivacaine desensitization of the area occurs after 5 to 10 min and lasts for 8 to 12 h [3]. Levobupivacaine (LB), the active l-isomer of bupivacaine, has similar effects to racemic bupivacaine but is less cardiotoxic [9].

Alpha-2 agonists, including dexmedetomidine (DEX), have been studied as adjuvants to LA to prolong the effect of regional blocks. The combination of LB and DEX was reported to produce 12 to 28 h of sensory block of the brachial plexus in dogs [10]. Perineural administration of DEX added to ropivacaine for sciatic nerve block prolonged the duration of analgesia [11] and caused a dose-dependent increase in the duration of thermal antinociception [12] in rats. The onset time was shortened, and the duration of the axillary brachial plexus block and the duration of postoperative analgesia were prolonged when DEX was added to LB in humans [13]. Dexmedetomidine administered intravenously (IV) at a dose of 1 µg/kg/h significantly prolonged sensory block after intrathecal administration of bupivacaine and morphine in dogs undergoing various hind leg orthopedic procedures [14].

Dexmedetomidine is an active d-isomer of the racemic mixture medetomidine. In dogs, its adverse effects are dose-dependent and mainly hemodynamic and include hypertension and bradycardia [15]. The hemodynamic effects are the result of the activation of pre- and postsynaptic α2-adrenergic receptors, followed by vasoconstriction, sympatholysis, and baroreflex-mediated parasympathetic activation [16]. 

The pharmacokinetic properties of DEX following IV administration in dogs are well known. After a single IV administration 20 μg/kg DEX, the volume of distribution (V_D_) was 0.86 ± 0.22 L/kg, the half-life (t_1/2_) was 0.78 ± 0.23 h, and the clearance (Cl) was 1.24 ± 0.48 L/kg/h. After a single IV application of 10 μg/kg, V_D_ was 0.79 ± 0.19 L/kg, t_1/2_ was 0.66 ± 0.18 h, and Cl was 0.97 ± 0.33 L/kg/h [17]. The mean maximum concentration (C_max_) for the IV dose (5 μg/kg), when extrapolated to the time of administration, was 18.6 ± 3.3 ng/mL and the time to reach maximal concentration (T_max_) was 1.5 ± 0.6 min. The half-life was 36 ± 6 min and the time at which DEX was still detectable in plasma (T_last_) was 110 ± 15 min. The clearance was 8.0 ± 1.6 mL/min/kg and V_D_ was 371 ± 72 mL/kg [18]. The pharmacokinetics of DEX as an adjunctive analgesic drug to regional anesthesia of the oral cavity with LB have not yet been studied. 

The primary aim of this study was to investigate the cardiopulmonary effects of DEX administered perineurally as an adjunctive analgesic to regional anesthesia of the upper and lower jaw with levobupivacaine in anesthetized dogs. The secondary aim of the study was to support the explanation of changes in cardiopulmonary parameters after DEX administration with the results of pharmacokinetic analysis. We hypothesized that infraorbital administration of DEX would result in more rapid and extensive absorption compared with inferior alveolar block due to an increased pressure gradient force across the capillary wall after drug administration in infraorbital canal. Therefore, a greater increase in arterial pressure and more pronounced bradycardia were expected with infraorbital block compared to inferior alveolar block.

## 2. Materials and Methods

The main and pilot studies were reviewed and approved by the Institutional Ethics Committee for Animal Welfare of the Veterinary Faculty, University of Ljubljana (No. 8-10-2020/7, date of approval 7 January 2021), and formal written consent was obtained from dog owners before participation in the study.

### 2.1. Pilot Study and Sample Size

A pilot study to determine the sample size was performed on six anesthetized client-owned dogs which underwent dental procedures (Table 1). All dogs received a comprehensive oral exam with full-mouth dental charting and dental radiographs, followed by basic periodontal therapy performed with a sonic scaler.

The primary endpoint was to determine the change in heart rate after perineural or IV administration of DEX. In all six dogs, levobupivacaine (Chirocaine 5 mg/mL, AbbVie, North Chicago, IL, USA) was administered perineurally immediately after performing full-mouth charting and dental radiographs as part of the standard anesthetic protocol to desensitize all four quadrants of the oral cavity. The dose of LB was calculated as b.w.^0.67^ × 0.11 mL/kg for the infraorbital block and b.w.^0.67^ × 0.18 mL/kg for the inferior alveolar block [3]. Dexmedetomidine (Dexdomitor, Zoetis, Parsippany-Troy Hills, NJ, USA) 0.5 µg/kg was administered together with LB into the left or right infraorbital canal (*n* = 2), into the submucosa at the opening of the left or right mandibular foramen (*n* = 2), or IV immediately after the application of LB (*n* = 2). Heart rate decreased by 30% after IV administration of DEX and by up to 20% after perineural administration in both infraorbital and inferior alveolar blocks. Based on the difference in heart rate decrease, we determined that at least five dogs per group (DEX IV, DEX infraorbital block, DEX inferior alveolar block, placebo IV) would be required for an alpha of 0.05 and a study power of 0.8. At least 10 dogs per group would be required, for a total of 40 dogs, for a study power of 1.0. G*Power (Heinrich-Heine University, Düsseldorf, Germany) was used to compute the statistical power analysis.

### 2.2. Main Study

A total of 40 dogs scheduled for a dental procedure at the Small Animal Clinic of the Veterinary Faculty, University of Ljubljana were studied. All dogs received a comprehensive oral exam with full-mouth dental charting and dental radiographs, followed by basic periodontal therapy performed with a sonic scaler.

Dogs that were in physical status I or II according to the American Society of Anesthesiologists assessment were considered eligible. A physical examination, complete blood count, and biochemical profile were performed on all dogs. Exclusion criteria included brachycephalic dog breeds; history of endocrinologic, cardiovascular, renal, or hepatic disease; body weight less than 10 kg; and ongoing therapy with angiotensin-converting enzyme inhibitors, corticosteroids, or nonsteroidal anti-inflammatory drugs.

**Figure 1:**
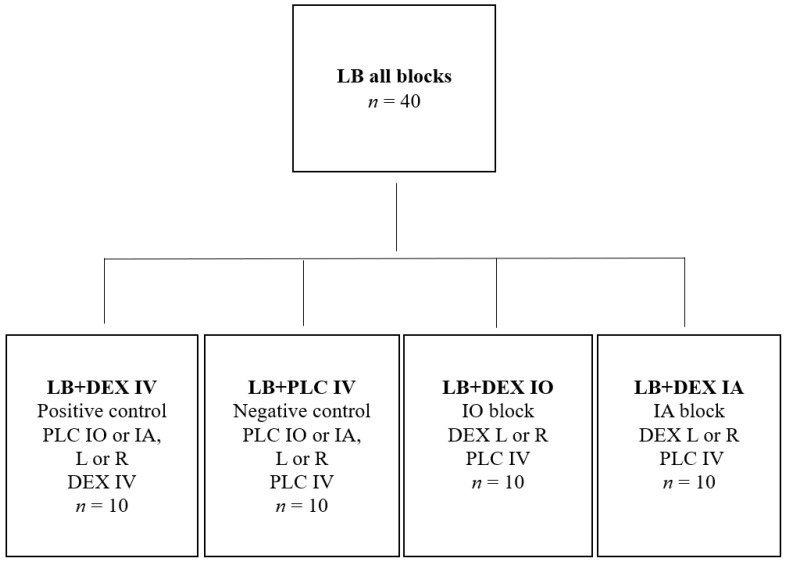
Dogs were randomly assigned to four groups of 10 animals. All dogs were injected with levobupivacaine (all blocks). Dexmedetomidine 0.5 µg/kg was administered into the infraorbital canal (LB + DEX IO), submucosally near the inferior alveolar nerve (LB + DEX IA) or intravenously (LB + DEX IV). The placebo IV (LB + PLC IV) group was administered Sterile Water for Injection IV. The dose of levobupivacaine was calculated as b.w.^0.67^ × 0.11 mL/kg for the infraorbital block and b.w.^0.67^ × 0.18 mL/kg for the inferior alveolar block. The volume of perineural or IV placebo was equal to the volume of dexmedetomidine. DEX, dexmedetomidine; IA, inferior alveolar block; IO, infraorbital block; IV, intravenously; L, left; LB, levobupivacaine; PLC, placebo (Sterile Water for Injection); R, right.

The dogs were randomly assigned (Table S1) to four groups of 10 dogs using a commercially available program (Research Randomizer, https://www.randomizer.org/, accessed on 11 January 2021, Urbaniak, G. C., & Plous, S. (2013). Research Randomizer (Version 4.0) [Computer software]) (Figure 1). 

All dogs received LB to desensitize all four quadrants of the oral cavity (left and right infraorbital block, left and right inferior alveolar block). The dose of LB was calculated as in the pilot study. The positive control group (LB + DEX IV) received DEX 0.5 µg/kg IV diluted to 1 mL with Sterile Water for Injection (B Braun Medical Inc., Bethlehem, PA, USA), and the negative control group (LB + PLC IV) received IV 1 mL of placebo (Sterile Water for Injection, B Braun Medical Inc., Bethlehem, PA, USA). Both positive and negative control groups also received a placebo perineurally together with LB in one of the blocks. The volume (0.2 mL) of perineural placebo in the control groups was equal to the volume of perineural DEX in the test groups. Dexmedetomidine (500 µg/mL) was diluted 1:5 with water for injection to allow accurate dosing. Water for injection was added to the calculated dose of DEX to a final volume of 0.2 mL. The first test group (LB + DEX IO) received DEX 0.5 µg/kg in the left or right infraorbital block and 1 mL placebo IV, while the second test group (LB + DEX IA) received DEX 0.5 µg/kg in the left or right inferior alveolar block and 1 mL placebo IV. The syringe containing the drugs for perineural administration (LB with DEX in the test groups and LB with placebo in the control groups) was always administered as the last block. The stomatologist (A.N.) who administered the blocks and the anesthesiologist (M.P.) who performed the anesthesia and post-procedure evaluations did not know which group each dog was assigned to until the end of the study (double-blind study). For this reason, all injections were always prepared in a different room and by a different anesthesiologist who also participated in the study (A.S.).

Food was withheld for no more than 12 h before anesthesia and water were allowed ad libitum until the procedure. For drug administration, a 20-gauge catheter (BD Venflon, Becton Dickinson Infusion Therapy, Stockholm, Sweden) was aseptically placed in a cephalic vein. The dogs were IV premedicated with methadone 0.2 mg/kg (Comfortan, Dechra, Northwich, UK) and 5 min later induced to anesthesia with propofol (Propoven, Fresenius Kabi, Lake Zurich, IL, USA) titrated until endotracheally intubated. Anesthesia was maintained with isoflurane (Isoflurin, Vetpharma Animal Health, Barcelona, Spain) in oxygen (FiO_2_ = 1) delivered through a circle breathing system (22 mm Flextube breathing system; Intersurgical Ltd., Wokingham, UK). The dogs breathed spontaneously during anesthesia. They were placed in dorsal recumbency on a dentistry table covered with soft padding, and forced-air warming (Bair Hugger, 3M, St. Paul, MN, USA) was used when rectal temperature decreased below 37 °C.

**Figure 2:**
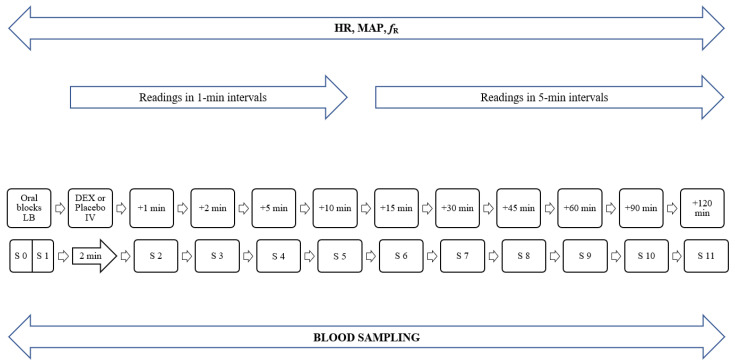
Timeline of events. DEX, dexmedetomidine; LB, levobupivacaine; IV, intravenous; MAP, mean arterial pressure; HR, heart rate; *f*_R_, respiratory rate; S, sample; S 0, sample taken prior to administration of oral blocks (B, baseline values); S 1, sample taken immediately after the completion of the last block (BL).

A 22-gauge catheter was aseptically placed in the dorsal pedal artery (BD Venflon, Becton Dickinson Infusion Therapy, Stockholm, Sweden) for direct arterial pressure measurement. Systolic (SAP), diastolic (DAP), and mean (MAP) arterial pressures; HR; respiratory rate (*f*_R_); end-tidal isoflurane concentration (FE’Iso); end-tidal CO_2_ tension (PE’CO_2_); ECG (lead II); and rectal temperature were monitored continuously with a multiparameter monitor (B105 Patient Monitor, GE Medical Systems Information Technologies Inc, Milwaukee, WI, USA). The measurements of SAP, DAP, MAP, HR, and *f*_R_ were recorded three times within 15 s to minimize the possibility of recording errors at each measurement point. The basal values were recorded immediately after the arterial catheter was placed. The oral blocks were performed and then either DEX or placebo was administered IV over two min. The above parameters were recorded every min for the first 12 min after the start of IV administration of DEX or placebo, and every 5 min thereafter until the end of the procedure (Figure 2). 

Both infraorbital blocks were performed with an intraoral technique using a 42 mm 27-gauge needle (Sulzer Mixpac GmbH, Kiel, Germany). The length to which the needle was introduced into the infraorbital canal was measured from the distal root of the ipsilateral third premolar tooth to the junction of the zygomatic bone with the maxilla [3]. Both inferior alveolar blocks were performed with an extraoral technique, also using a 42 mm 27-gauge needle. First, the mandibular foramen was located and palpated on the medial aspect of the mandible, caudal and ventral to the ipsilateral last molar tooth. The needle was then advanced perpendicularly on the most ventral surface of the mandible and guided medially as close to the bone as possible towards the foramen [3]. With both techniques, the bevel of the needle was always oriented in the same direction as the nerve fibers, and negative pressure was always applied to the syringe before the injection of drugs. All blocks were performed in an aseptic manner and by the same stomatologist (A.N.).

Carprofen 4 mg/kg IV (Rycarfa, Krka, Novo mesto, Slovenia) was administered to all dogs during the procedure when SAP was at least 80 mmHg. A bolus of fentanyl (2 µg/kg IV; Fentanyl Torrex, Chiesi, Parma, Italy) was administered as a rescue analgesic when HR, *f*_R_, or MAP increased by more than 30%. Propofol (0.5 to 1 mg/kg IV) was administered, and isoflurane setting was increased by 0.1% to deepen anesthesia when the positive palpebral reflex was observed. During the procedure, Ringer’s lactate solution (B Braun Medical Inc., Bethlehem, PA, USA) was infused with an infusion pump (Infusion Pump SK-600I Vet, Shenzhen Mindray Scientific Co. LTD, Shenzhen, China) at a rate of 5 mL/kg/hour. After the procedure, intravenous fluid therapy was administered at a rate of 2 to 6 mL/kg/hour until discharge.

At the end of the procedure, methadone 0.2 mg/kg was administered IV and repeated every 4 h until discharge if necessary, or a single IV dose of buprenorphine (0.02 mg/kg; Bupredine, Dechra, Northwhich, UK) was administered. If the procedure was considered to be very painful or very invasive (e.g., multiple tooth extractions, full-mouth gingivectomy), the analgesia was reinforced with a transdermal fentanyl patch of 4 µg/kg/h (Durogesic, Janssen, Titusville, NJ, USA). The dogs were prescribed peroral carprofen 4 mg/kg (Rimadyl, Pfizer, New York, NY, USA) for 2 to 7 days.

### 2.3. Blood Sampling

Blood (4 mL) was collected via a dorsal pedal artery catheter into tubes containing lithium heparinate (BD Vacutainer, Becton Dickinson and Company, Franklin Lakes, NJ, USA) prior to administration of oral blocks (B, baseline values), immediately after administration of oral blocks (BL), and 1, 2, 5, 10, 15, 30, 45, 60, 90, and 120 min after completion of IV application of DEX or placebo (Figure 2). The time points of blood sampling (up to 120 min after administration of DEX) were determined according to previously published data on the pharmacokinetics of DEX administered IV (T_last_ 110 ± 15 min after a 10-fold higher dose than that in our study) [18]. For each collection, 1 mL of blood from the catheter was discarded, the sample collected, and the system flushed with 4 mL of 0.9% Sodium Chloride Solution (B Braun Medical Inc., Bethlehem, PA, USA). A different syringe was used for each step (3-syringe technique). Blood samples were centrifuged immediately after collection at 1500 g for 15 min at room temperature. Plasma samples were separated into aliquots and stored at −80 °C until analysis. 

### 2.4. Laboratory Analysis

Plasma samples were prepared for analysis using an Ostro^TM^ 96-well plate (Waters Corp., Milford, MA, USA) for protein precipitation and phospholipid removal. Internal standard DEX-d4 (10 µL, Dexmedetomidine-d4 L-Tartrate, LGC/Toronto Research Chemicals, Canada) at a concentration of 10 ng/mL was added to a 50-µL plasma sample and diluted to a volume of 150 µL with 0.1% formic acid. This mixture was transferred to the 2-mL well of the Ostro^TM^ well plate before adding 450 μL of 0.1% formic acid in acetonitrile. Samples were then aspirated three times with a multichannel pipette and placed onto a positive pressure processor (Waters Corp., Milford, MA, USA) by setting the flow to 60 psi for 5 min. The eluate was dried under a gentle stream of nitrogen and then reconstituted in a 1 mL acetonitrile/0.1% formic acid mixture (3/7). Finally, the sample was filtered through a 0.2 µm regenerated cellulose membrane syringe filter (Phenomenex, Torrance, CA, USA) and analyzed by liquid chromatography coupled to tandem mass spectrometry (LC-MS/MS).

LC-MS/MS analysis was performed using a Nexera ultra high-performance LC (Shimadzu Corp., Japan) coupled with a QTRAP^®^ 4500 MS/MS system (AB Sciex, Framingham, MA, USA). Separation was achieved at room temperature using a 5 cm Ascentis Express C18 (Supelco, Bellefonte, PA, USA) column with a 2 µm particle size and 2.1 mm internal diameter. The mobile phases were acetonitrile (A) and 0.1% formic acid (B). The flow rate was 0.3 mL/min and the injection volume was 20 µL. The gradient started at 70% B and was ramped down to 50% B in 2.5 min In the next 0.5 min, it was ramped down again to 0% B and then held for the next 0.5 min. Finally, it was ramped back up to 70% B and held for 1.5 min to allow the column to equilibrate. The mass spectrometer was operated under positive electrospray ionization at a source temperature of 600 °C and in multiple reaction monitoring acquisition mode. The operating software for the data station was Analyst v1.6.3. For DEX, the 201 > 95, 201 > 115, and 201 > 91 transitions were monitored, with the first used as the quantifying transition. For the internal standard DEX-d4, transition 205 > 99 was used to control the retention time and process quality.

The calibration range was from 0.02 to 10 ng/mL, with a linear regression quotient > 0.99. The instrumental limit of quantification (LOQ) was 0.02 ng/mL and the limit of detection (LOD) was 0.002 ng/mL, corresponding to 0.4 ng/mL and 0.04 ng/mL at the method level, respectively. The quality of sample preparation and analysis was monitored by solvent blanks, process blanks, and quality control samples prepared by spiking the blank plasma with the analyte and the internal standard at the LOQ level. All quality control procedures were in accordance with FDA guidelines for the validation of bioanalytical methods (https://www.fda.gov/files/drugs/published/Bioanalytical-Method-Validation-Guidance-for-Industry.pdf, accessed on 1 October 2021).

### 2.5. Pharmacokinetic Analysis

Standard pharmacokinetic parameters, plasma elimination half-time (t_1/2_), plasma clearance rate (CL_p_), and volume of distribution (V_D_) were calculated by non-compartmental analysis of actual plasma concentration–time curves using non-linear regression two-phase elimination compared to one-phase elimination using GraphPad Prism version 9.00 for Mac, GraphPad Software, La Jolla California USA (www.graphpad.com, accessed on 16 December 2021). 

Plasma elimination half-life (t_1/2_) was calculated by the equation,

t_1/2_ = 0.693/k_el_, (1)

where k_el_ is the elimination rate constant. 

Plasma clearance (CL_p_) was calculated by the equation,

CL_p_ = dose/AUC_(0__→120 min)_ (2)

Volume of distribution (V_D_) was calculated by the equation,

V_D_ = (t_1/2_ × CLp)/ln2 (3)

The area under the curve (AUC_(0__→120 min)_) was calculated as the AUC from the time 0 to the last quantifiable data point (120 min) using a linear trapezoidal method. The data are presented as mean ± standard error of the mean (SEM).

### 2.6. Statistical Analysis

Statistical analysis was performed using Jamovi 2.0.0.0 (The Jamovi Project 2021, Australia) and RStudio (RStudio Team (2021). RStudio: Integrated Development for R. RStudio, PBC, Boston, MA URL http://www.rstudio.com/, accessed on 16 December 2021). After the normality of distribution was analyzed using the Shapiro–Wilk test, physiological variables within groups were compared with baseline values. Normally distributed variables were compared using the paired t-test, whereas non-normally distributed variables were compared with the Wilcoxon rank test. Because of a mixture of normal and non-normal data, values are expressed as the median and interquartile range. Differences between groups were analyzed using a one-way ANOVA with Tukey’s multiple comparison post-hoc test and appropriate adjustment for multiple testing. In cases where the assumption of normality or homogeneity of variances was violated, the Kruskal–Wallis test was used with Dwass–Steel-Critchlow–Fligner (DSCF) post-hoc multiple comparison analysis. Partial eta-squared (η_p_^2^) was calculated as a measure of effect size. A generalized linear model with mixed effects was fitted to determine the effect of DEX on physiological variables. The fixed effect was the concentration of DEX and the random effect was the dogs. Statistical significance was set at *p* < 0.05.

## 3. Results

The study was completed successfully in all dogs with no complications. 

The dogs in the pilot study were 101.00 ± 44.14 months old (mean ± SD) and weighed 21.37 ± 9.51 kg (mean ± SD), and the dogs in the main study were 59.15 ± 42.52 months old and weighed 25.21 ± 9.79 kg. Dental procedures in the main study are presented in Table 2.

None of the dogs in the main study required rescue analgesia during anesthesia. A propofol bolus was administered and/or isoflurane setting was increased to deepen anesthesia in three dogs in the LB + DEX IV group, six dogs in the LB + DEX IO group, seven dogs in the LB + DEX IA group and six dogs in the LB + PLC IV group.

The volume of blood collected for pharmacokinetic analysis (4 mL × 12 = 48 mL) plus the volume of blood discarded from the catheter during collection (1 mL × 12 = 12 mL) totaled 60 mL. This corresponded to 2.75% of the total blood volume in the LB + DEX IV group (24.24 ± 12.25 kg, mean weight ± SD), 2.64% of the total blood volume in the LB + PLC IV group (25.23 ± 6.43 kg), 2.66% of the total blood volume in the LB + DEX IO group (25.09 ± 12.61 kg), and 2.64% of the total blood volume in the LB + DEX IA group (25.26 ± 8.40 kg). In the smallest dog in this study, which weighed 10.8 kg, 60 mL of blood accounted for 6.2% of the total blood volume. The blood loss due to the dentistry procedure was estimated to be no more than 3% in any dog. Before and after the procedure, PCV was checked, and the values were within the reference range in all dogs.

### 3.1. Pharmacokinetic Analysis

**Figure 3:**
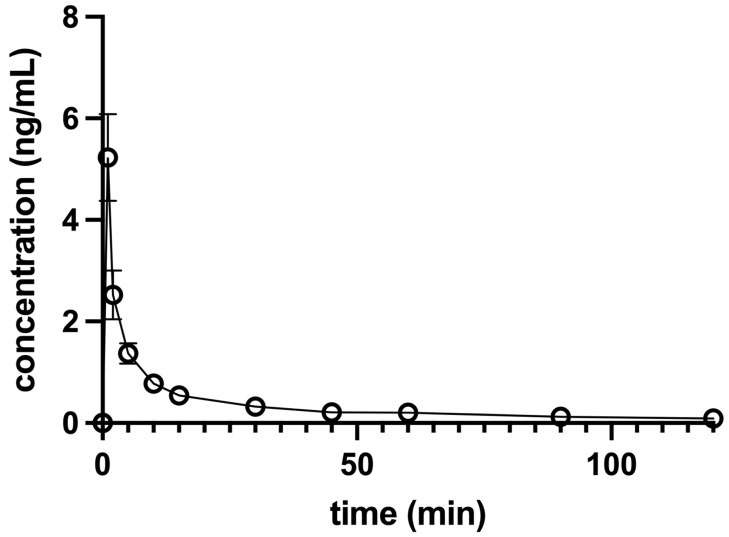
Concentration-versus-time profile of a single 0.5 µg/kg intravenous dose of dexmedetomidine administered to 10 anesthetized dogs. Dogs were premedicated with methadone intravenously, and anesthesia was induced with propofol and maintained with isoflurane in oxygen. Levobupivacaine was used for both infraorbital and inferior alveolar blocks. The data are presented as mean ± SEM.

Dexmedetomidine plasma concentrations versus the time of each dog and the combined data of all 10 dogs in a group were analyzed. The maximum concentration (C_max_) after IV application of 0.5 µg/kg DEX was 7.29 + 0.13 ng/mL when extrapolated to the time of administration (T_0_) and 5.23 ± 0.85 ng/mL when determined from raw data. The elimination curve showed an exponential decline, which was best fitted to one-phase elimination with a t_1/2_ of 5.98 ± 0.46 min (Figure 3).

**Figure 4:**
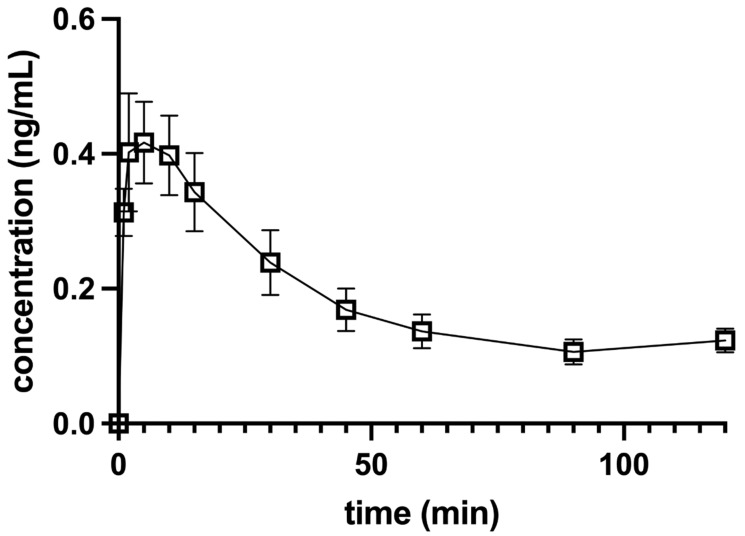
Concentration-versus-time profile of a single 0.5 µg/kg dose of dexmedetomidine injected together with levobupivacaine perineurally into the left (*n* = 5) or right (*n* = 5) infraorbital canal in 10 anesthetized dogs. Dogs were premedicated with methadone intravenously, and anesthesia was induced with propofol and maintained with isoflurane in oxygen. Levobupivacaine was used for both infraorbital and inferior alveolar blocks. The data are presented as mean ± SEM.

**Figure 5:**
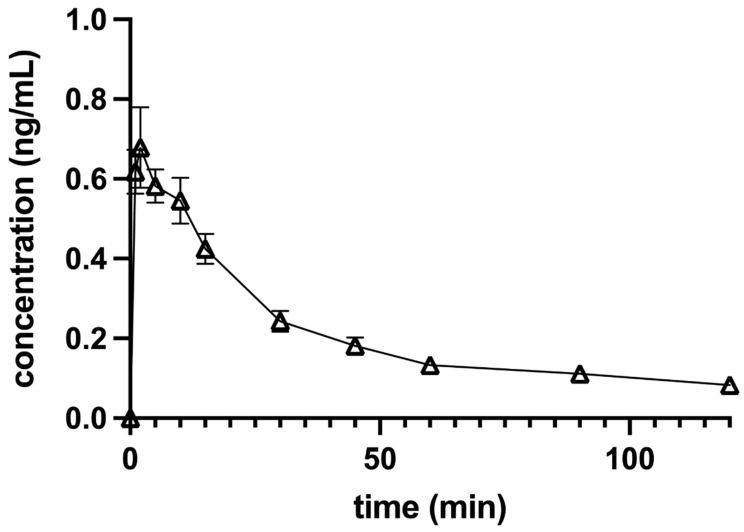
Concentration-versus-time profile of a single 0.5 µg/kg dose of dexmedetomidine injected together with levobupivacaine perineurally into the submucosa at the opening of the left (*n* = 5) or right (*n* = 5) mandibular foramen in 10 anesthetized dogs. Dogs were premedicated with methadone intravenously, and anesthesia was induced with propofol and maintained with isoflurane in oxygen. Levobupivacaine was used for both infraorbital and inferior alveolar blocks. The data are presented as mean ± SEM.

The C_max_ after IO perineural application of 0.5 µg/kg DEX was 0.47 ± 0.08 ng/mL at a T_max_ (time to reach maximum concentration) of 7.22 ± 1.28 min (Figure 4), and the C_max_ after IA perineural application was 0.76 ± 0.09 ng/mL at a T_max_ of 7.50 ± 1.63 min (Figure 5). The mean terminal-phase t_1/2_ was 63.44 ± 24.15 min for IO and 23.78 ± 3.78 min for IA DEX administration. Both were significantly longer (*p* < 0.01) compared to IV administration. 

The bioavailability of DEX was 0.48 for the IO block and 0.56 for IA administration. The other calculated pharmacokinetic parameters are listed in Table 3. 

### 3.2. Pharmacodynamic Analysis

To verify that the groups had the same characteristics and did not differ with respect to possible confounding factors, such as weight and age, the Kruskal–Wallis test was performed. No significant differences were found between all four groups with respect to weight H(3) = 2.106, *p* = 0.55, and age H(3) = 1.028, *p* = 0.80. In terms of HR (F(3) = 0.324, *p* = 0.81), MAP (F(3) = 1.498, *p* = 0.23), and *f*_R_ (H(3) = 1.162, *p* = 0.76), there were no significant differences between groups before the administration of DEX.

**Figure 6:**
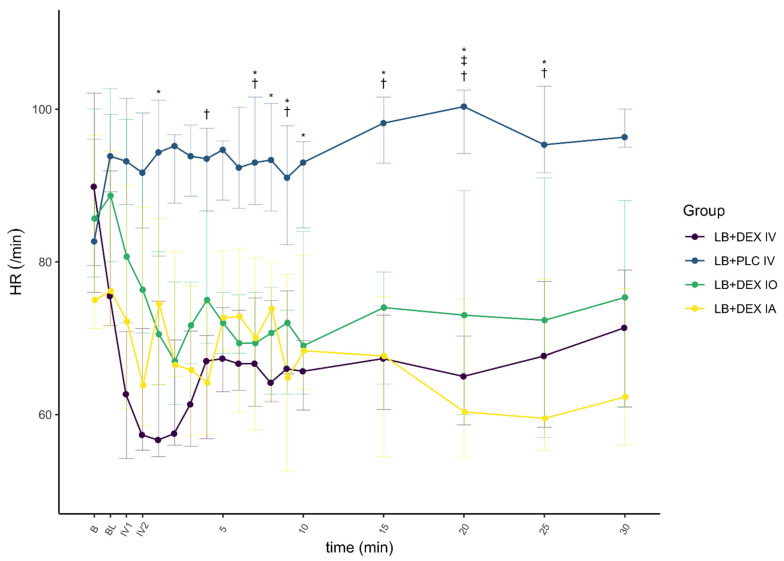
Heart rate (HR) before and after administration of dexmedetomidine (DEX) and/or placebo with time points (5, 10, 15, 20, 25 and 30 min) presenting values after completion of IV application of DEX or placebo. Levobupivacaine was used for both infraorbital and inferior alveolar blocks. Values are expressed as median and IQR. B, baseline value, prior to administration of oral blocks; BL, immediately after administration of oral blocks; HR, heart rate; IV1, after one min/completion of IV application of DEX or placebo; IV2, after two min/completion of IV application of DEX or placebo; LB + DEX IA, dogs (*n* = 10) which received levobupivacaine and dexmedetomidine perineurally into the submucosa at the opening of the mandibular foramen; LB + DEX IO, dogs (*n* = 10) which received levobupivacaine and dexmedetomidine perineurally into the infraorbital canal; LB + DEX IV, dogs (*n* = 10) which received levobupivacaine perineurally and dexmedetomidine IV (positive control); LB + PLC IV, dogs (*n* = 10) which received levobupivacaine perineurally and placebo IV (negative control). * significant difference (*p* < 0.05) between the LB + PLC IV and LB + DEX IV groups. † significant difference (*p* < 0.05) between the LB + PLC IV and LB + DEX IA groups. ‡ significant difference (*p* < 0.05) between the LB + PLC IV and LB + DEX IO groups.

**Figure 7:**
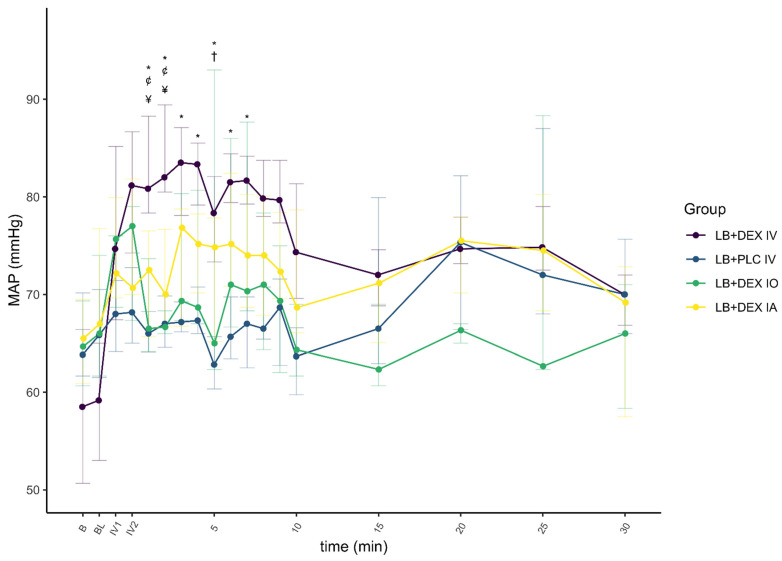
Mean arterial pressure (MAP) before and after administration of dexmedetomidine (DEX) and/or placebo with time points (5, 10, 15, 20, 25 and 30 min) presenting values after completion of IV application of DEX or placebo. Levobupivacaine was used for both infraorbital and inferior alveolar blocks. Values are expressed as median and IQR. B, baseline value, prior to administration of oral blocks; BL, immediately after administration of oral blocks; IV1, after one min/completion of IV application of DEX or placebo; IV2, after two min/completion of IV application of DEX or placebo; LB + DEX IA, dogs (*n* = 10) which received levobupivacaine and dexmedetomidine perineurally into the submucosa at the opening of the mandibular foramen; LB + DEX IO, dogs (*n* = 10) which received levobupivacaine and dexmedetomidine perineurally into the infraorbital canal; LB + DEX IV, dogs (*n* = 10) which received levobupivacaine perineurally and dexmedetomidine IV (positive control); LB + PLC IV, dogs (*n* = 10) which received levobupivacaine perineurally and placebo IV (negative control); MAP, mean arterial pressure. * significant difference (*p* < 0.05) between the LB + PLC IV and LB + DEX IV groups. † significant difference (*p* < 0.05) between the LB + PLC IV and LB + DEX IA groups. ¢ significant difference (*p* < 0.05) between the LB + DEX IV and LB + DEX IA groups. ¥ significant difference (*p* < 0.05) between the LB + DEX IV and LB + DEX IO groups.

**Figure 8:**
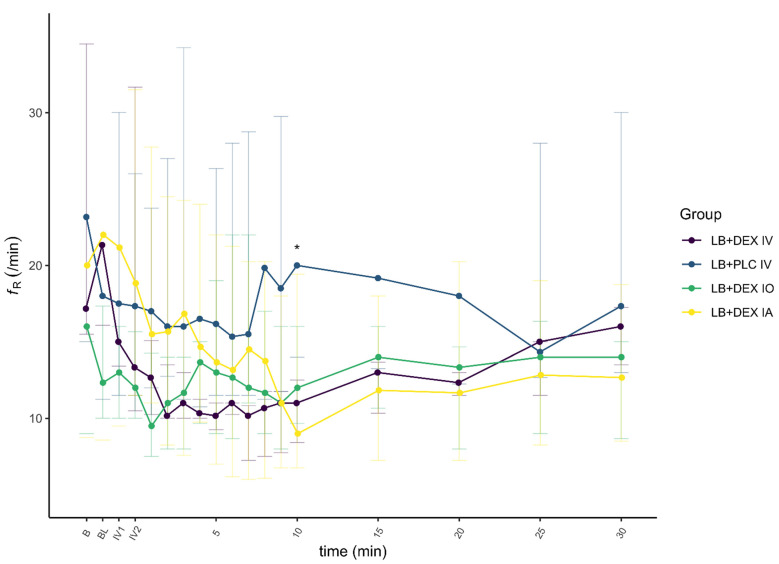
Respiratory rate (*f*_R_) before and after administration of dexmedetomidine (DEX) and/or placebo. Time points (5, 10, 15, 20, 25 and 30 min) present values after completion of IV application of DEX or placebo. Levobupivacaine was used for both infraorbital and inferior alveolar blocks. Values are expressed as median and IQR. B, baseline value, prior to administration of oral blocks; BL, immediately after administration of oral blocks; *f*_R,_ respiratory rate; IV1, after one min/completion of IV application of DEX or placebo; IV2, after two min/completion of IV application of DEX or placebo; LB + DEX IA, dogs (*n* = 10) which received levobupivacaine and dexmedetomidine perineurally into the submucosa at the opening of the mandibular foramen; LB + DEX IO, dogs (*n* = 10) which received levobupivacaine and dexmedetomidine perineurally into the infraorbital canal; LB + DEX IV, dogs (*n* = 10) which received levobupivacaine perineurally and dexmedetomidine IV (positive control); LB + PLC IV, dogs (*n* = 10) which received levobupivacaine perineurally and placebo IV (negative control). * significant difference (*p* < 0.05) between the LB + PLC IV and LB + DEX IV groups.

There was a significant effect of DEX on HR (F(1) = 29.992, *p* < 0.01, ηp2 = 0.08) and MAP (F(1) = 24.663, *p* < 0.01, ηp2 = 0.06), and a smaller effect on *f*_R_ (F(1) = 12.726, *p* < 0.01, ηp2 = 0.03). The changes in cardiovascular parameters and *f*_R_ associated with the control and test groups are summarized in Figure 6, Figure 7 and Figure 8.

Heart rate in the LB + DEX IV group was significantly lower from the first minute of IV administration (IV1) to 30 min after the end of IV administration compared to baseline. In the LB + PLC IV group, HR was significantly higher only at IV1. Heart rate in the LB + DEX IO group was significantly lower than baseline from the second min of IV administration (IV2) to 30 min after the end of IV administration. Heart rate in the LB + DEX IA group was significantly lower at IV1 and IV2 and 2 to 4, 9, and 15 to 30 min after the end of IV administration. Five dogs in the LB + DEX IV group and one dog each in the LB + DEX IO and LB + DEX IA groups developed second-degree atrioventricular (AV) blocks either during IV administration or up to one min after the end of IV administration.

In the LB + DEX IV group, there was a significant increase in MAP from IV1 to 30 min after the end of IV administration compared to baseline. In the LB + PLC IV group, there were no significant changes in MAP. In the LB + DEX IO group, MAP was significantly higher at IV1, IV2, and 2 to 5, 7, and 8 min after the end of IV administration. Except for 8 min after the end of IV administration, there was a significant increase in MAP in the LB + DEX IA group from IV1 to 9 min after the end of IV administration. The LB + DEX IV group showed a significant decrease in *f*_R_ from 1 to 15 min after the end of IV administration. The *f*_R_ of the LB + PLC IV group was not significantly different from the baseline during the procedure. The LB + DEX IO group showed a significant decrease in *f*_R_ from 2 to 4 min after the end of IV administration. In the LB + DEX IA group, *f*_R_ was significantly lower 3 min and 8 to 20 min after the end of IV administration.

## 4. Discussion

This randomized double-blinded interventional clinical study investigated the pharmacokinetics and effects of DEX on hemodynamics and respiration when administered together with LB into the infraorbital canal to desensitize the infraorbital nerve or into the submucosa at the opening of mandibular foramen to desensitize the inferior alveolar nerve and compared it to IV administration.

Perineural administration of DEX resulted in a lower C_max_ and shorter T_max_ in the LB + DEX IO group compared to the LB + DEX IA group. In dogs with a bodyweight of 14.6 ± 1.5 kg, the width and height of the infraorbital canal at the narrowest point were 3.1 ± 0.2 mm and 3.6 ± 0.2 mm, respectively [19]. The volume of LB used in a 15 kg dog in our study was 0.68 mL. The DEX concentration was 500 µg/mL and had to be diluted for accurate dosing (7.5 µg in a 15 kg dog). The final volume of diluted DEX was 0.2 mL and was mixed with 0.68 mL of LB. Although the pressure within the IO canal was not measured, we presume that the application of drugs into the infraorbital canal increased the pressure within it and resulted in faster absorption in the LB + DEX IO group compared to the LB + DEX IA group. On the other hand, the higher C_max_, better bioavailability (higher AUC_(0__→120min)_), and faster elimination rate (almost three-times shorter t_1/2_) after IA application might be due to better vascularization and perfusion of the submucosa near the mandibular foramen.

After perineural administration of DEX, significantly longer t_1/2_ and lower AUC_(0__→120min)_ were observed compared to IV application. The process of absorption is a limiting factor in extravascular administration and the terminal t_1/2_ reflects the rate and extent of absorption and not the elimination process [20]. The bioavailability was 48% and 56% for IO and IA application, respectively, and was lower compared to intramuscular administration (60%) [21] but higher than that after oral transmucosal application (11.2% and 16.34%, respectively) [18,22].

Dexmedetomidine plasma concentration after the IV dose decreased exponentially. The high SEM of the pooled DEX concentration data determined one min after injection reflects the overlap of the distribution and elimination phases of DEX pharmacokinetics, while at 5 min elimination already predominated and the SEM stabilized. Dexmedetomidine may reduce its own elimination dose-dependently as observed in healthy human volunteers, mainly due to the decrease in cardiac output [23] resulting in decreased hepatic blood flow and slower metabolization rate. A prolonged t_1/2_ is expected with higher doses and overdoses or when DEX is co-administered with drugs that affect hepatic circulation [21]. The dogs in our study were anesthetized with isoflurane which dose-dependently decreases cardiac output [24]. Dexmedetomidine may counterbalance the effects of isoflurane in terms of vasodilatation and stabilize vascular tone [25]. On the other hand, isoflurane may attenuate the increase of systemic vascular resistance induced by DEX [26,27]. Cardiac output was not measured in our study, but the relatively short t_1/2_ and high clearance compared to 10- [18] to 40-fold [17] higher doses used in non-anesthetized dogs suggest that hepatic blood flow may not have been reduced because of the positive hemodynamic interaction between the low dose of DEX and isoflurane.

The V_D_ of DEX in our study was lower compared to that reported with 10- [18] to 40-fold [17] higher doses of DEX and reflects a negligible distribution of DEX from the central to the peripheral compartment. Dexmedetomidine is a lipophilic drug with a high degree of protein binding; 94% of DEX is bound to albumin and α_1_-glycoprotein in plasma [16]. The calculated V_D_ indicates that a low dose of DEX is distributed mainly in plasma and extracellular fluid. The calculated AUC_(0__→120min)_ for IV administered DEX in our study was 42.11 ± 5.01 ng/mL × min. In dogs given DEX at a 10-fold higher dose, the AUC_(0__→inf)_ was 655 ± 148 ng/mL × min [18], which indicates that plasma levels are directly proportional to the dose.

The cardiopulmonary response to DEX administration in our study was consistent with already published data [16,18,28]. In the LB + PLC IV group, no significant changes from baseline were observed for HR (except at time IV1), MAP, and *f*_R_. In all other groups, HR, MAP, and *f*_R_ changed significantly at different time points, with HR and *f*_R_ lower and MAP higher than baseline. In dogs anesthetized with isoflurane [16] and sevoflurane [28], a decrease in HR and an increase in blood pressure were reported after a DEX IV loading dose of 0.5 µg/kg. Decreased HR and *f*_R_ were also reported in non-anesthetized dogs administered DEX IV at a 10-fold higher dose [17]. The frequency of second-degree AV blocks observed after administration of DEX in our study was proportional to its plasma concentration, i.e., they were observed more often in the LB + DEX IV group. These results are consistent with those of Kuusela et al. [29] and Congdon et al. [30], in which dogs developed second-degree AV blocks after intramuscular administration of 10 µg/kg DEX.

Dogs administered DEX IV or perineurally had a lower HR than those in the LB + PLC IV group, while a higher MAP was observed only in the LB + DEX IV and LB + DEX IA groups. Lower HR and higher systolic blood pressure were reported in dogs under sevoflurane anesthesia that received DEX IV 0.5 µg/kg compared to the control group [28]. The hemodynamic effects of DEX in dogs are a consequence of the activation of postsynaptic α_2_-receptors in peripheral vascular smooth muscle, resulting in peripheral vasoconstriction. The initial increase in blood pressure is associated with increased vagal tone and bradycardia [31]. Medetomidine, a racemic mixture of the two optical isomers levomedetomidine and dexmedetomidine, produced similar hemodynamic changes at doses of 1 to 20 µg/kg IV, although the changes were less evident at the lowest doses (1 to 2 µg/kg) [32].

The difference, although non-significant, in MAP and HR in the LB + DEX IV and LB + DEX IA groups compared to the LB + DEX IO group was due to a higher plasma concentration of DEX (more than 10-fold higher C_max_ after IV administration and more than 7-fold higher C_max_ after IA administration). The better bioavailability of DEX after IA administration may be attributed to greater absorption of DEX after application of the drug into the well-perfused submucosa in the vicinity of the mandibular foramen. The highest MAP and lowest HR and *f*_R_ in the LB + DEX IO group were observed earlier than in the LB + DEX IA group, consistent with the shorter, although not significant, T_max_ in the LB + DEX IO group. We presume that this may be due to an increased pressure gradient force across the capillary wall immediately after drug administration into the infraorbital canal. The early oscillations in cardiopulmonary parameters after application of oral blocks in all dogs were attributed to nociceptive stimulation due to the dental procedure and the time to onset of sensory blockade with LB (usually less than 15 min if applied close to the nerve) [33].

The present study has some limitations which need to be addressed. First, because this was a clinical study, the dogs underwent different dentistry procedures. Although blood samples were taken up to 120 min after IV application, the statistical analysis of cardiopulmonary parameters (HR, MAP, *f*_R_) and the comparison between groups was performed only up to 30 min after IV application of DEX or placebo. According to the calculation of the power of the study, at least five dogs per group were required for α = 0.05 and a study power of at least 0.8. This requirement was met only up to 30 min after IV application because in some dogs the procedure was terminated within 30 min. It would not be ethical to prolong anesthesia only because of the requirements of this research. Second, although all blood samples were collected for pharmacokinetic analysis from each dog, in some of them the procedure was completed before the last sampling. In this case, the remaining blood samples were collected from non-anesthetized dogs and this might affect the pharmacokinetics of DEX. Last, because of the repeated blood sampling (the total volume of blood withdrawn from each dog was 60 mL), no dogs weighing less than 10 kg were included in this study.

## 5. Conclusions

The hypothesis that the administration of DEX into the infraorbital canal would result in faster and more extensive absorption leading to a greater increase of MAP and more pronounced bradycardia compared to IA administration was not confirmed. Although IO administration resulted in faster absorption of DEX, the bioavailability was better and elimination faster at IA administration. No difference in MAP and HR was observed between the LB + DEX IO and LB + DEX IA groups; however, MAP was higher in the LB + DEX IV group when compared to perineural administration. Perineural administration of DEX was associated with lower plasma concentration compared to IV administration and may be preferable because it induces a less pronounced cardiovascular response in terms of bradycardia and hypertension. 

## Figures and Tables

**Table 1:** Dental procedures in the pilot study.

Group	Procedure
LB + DEX IV	EX of up to four teeth on R MAX, multi EX
LB + DEX IO	EX of up to four teeth on L MAN, EX of up to four teeth on L MAX
LB + DEX IA	2 × multi EX

EX, extraction; L, left; LB + DEX IA, dogs (*n* = 2) which received levobupivacaine and dexmedetomidine perineurally into the submucosa at the opening of the mandibular foramen; LB + DEX IO, dogs (*n* = 2) which received levobupivacaine and dexmedetomidine perineurally into the infraorbital canal; LB + DEX IV, dogs (*n* = 2) which received levobupivacaine perineurally and dexmedetomidine IV; MAN, mandibula; MAX, maxilla; multi EX, multiple extractions (five or more teeth extracted on all four quadrants); R, right.

**Table 2:** Dental procedures in the main study.

Group	Procedure
LB + DEX IV	2 × EX of up to four teeth on R MAX, 2 × EX of up to four teeth on L MAX, 3 × EX of up to four teeth on L MAN, multi EX, GG on L + R MAN, PCP of C on R MAN
LB + PLC IV	2 × EX of up to four teeth on L MAX, EX of up to four teeth on L MAN, 3 × multi EX, multi EX with full-mouth GG, PCP of C on L MAN, PCP of C on L + R MAN, ENDO of C on L MAX
LB + DEX IO	EX of up to four teeth on R MAX, 2 × EX of up to four teeth on L MAX, EX of up to four teeth on L + R MAX, EX of up to four teeth on L MAN, EX of up to four teeth on R MAN with IPP on L + R MAX, 2 × multi EX, PCP of C on L + R MAN, ENDO of C on L MAN
LB + DEX IA	EX of up to four teeth on R MAX, 2 × EX of up to four teeth on L + R MAX, EX of up to four teeth on L MAN, 2 x EX of up to four teeth on R MAN, 3 × multi EX, ENDO of C on L MAN

C, canine tooth; ENDO, endodontic procedure; EX, extraction; GG, gingivectomy; IPP, inclined plane placement; L, left; LB + DEX IA, dogs (*n* = 10) which received levobupivacaine and dexmedetomidine perineurally into the submucosa at the opening of the mandibular foramen; LB + DEX IO, dogs (*n* = 10) which received levobupivacaine and dexmedetomidine perineurally into the infraorbital canal; LB + DEX IV, dogs (*n* = 10) which received levobupivacaine perineurally and dexmedetomidine IV (positive control); LB + PLC IV, dogs (*n* = 10) which received levobupivacaine perineurally and placebo IV (negative control); MAN, mandibula; MAX, maxilla; multi EX, multiple extractions (five or more teeth extracted on all four quadrants); PCP, partial coronal pulpectomy; R, right.

**Table 3:** Calculated pharmacokinetic parameters (mean ± SEM) of dexmedetomidine 0.5 µg/kg administered intravenously (IV, *n* = 10), perineurally into the left or right infraorbital canal (IO, *n* = 10) or perineurally into the submucosa at the opening of the left or right mandibular foramen (IA, *n* = 10). Dogs were premedicated with methadone intravenously and anesthesia was induced with propofol and maintained with isoflurane in oxygen. Levobupivacaine was used for both infraorbital and inferior alveolar blocks.

	AUC_(0__→__120)_ (ng/mL min)	V_D_ (L/kg)	Cl (mL/min/kg)
IV	42.11 ± 5.01	0.12 ± 0.02	14.17 ± 1.58
IO	20.08 ± 3.82 *	NA	NA
IA	23.78 ± 3.78 *	NA	NA

AUC_(0_**_→_**_120)_, the area under the curve from time 0 to the last quantifiable data point (120 min); Cl, clearance; IA, inferior alveolar block; IO, infraorbital block; IV, intravenously; NA, not applicable; V_D_, volume of distribution.* Compared to IV administration (*p* < 0.01, one-way ANOVA).

## Data Availability

Data supporting the reported results can be sent to anyone interested by contacting the corresponding author.

## Supplementary Materials

The following is available online at https://www.mdpi.com/article/10.3390/ani12091217/s1, Table S1: CONSORT 2010 checklist of information to include when reporting a randomised trial.

## References

[1] Aguiar J., Chebroux A., Martinez-Taboada F., Leece E.A. (2014). Analgesic effects of maxillary and inferior alveolar nerve blocks in cats undergoing dental extractions. J. Feline Med. Surg..

[2] Benito J., Monteiro B., Lavoie A.-M., Beauchamp G., Lascelles B.D.X., Steagall P.V. (2016). Analgesic efficacy of intraperitoneal administration of bupivacaine in cats. J. Feline Med. Surg..

[3] Verstraete F.J.M., Lommer M.J.,  Arzi B., Verstraete F.J.M., Lommer M.J., Arzi B. (2020). Anaesthesia and Pain Management. Oral and Maxillofacial Surgery in Dogs and Cats.

[4] Cicirelli V., Lacalandra G.M., Aiudi G.G. (2021). The effect of splash block on the need for analgesia in dogs subjected to video-assisted ovariectomy. Vet. Med. Sci..

[5] Castejón-González A.C., Reiter A.M. (2019). Locoregional Anesthesia of the Head. Vet. Clin. Small Anim. Pract..

[6] Alaman M., González-Marrón A., Lorente C., Bonastre C., Laborda A. (2021). Description of an Ultrasound-Guided Transverse Approach to the Transversus Thoracis Plane Block and Evaluation of Injectate Spread in Canine Cadavers. Animals.

[7] Martinez-Taboada F., Sun T.C., Redondo J.I. (2021). Onset of Action of Bupivacaine Administered via Dural Puncture Epidural versus Spinal, Lumbosacral, and Sacrococcygeal Epidural Injections in Dogs: Randomised Clinical Trial. Animals.

[8] Cicirelli V., Debidda P., Maggio N., Caira M., Lacalandra G., Aiudi G. (2021). Ultrasound-Guided Funicular Block: Ropivacaine Injection into the Tissue around the Spermatic Cord to Improve Analgesia during Orchiectomy in Dogs. Animals.

[9] McLeod G.A., Burke D. (2001). Levobupivacaine. Anaesthesia.

[10] Campoy L., Read M.R. (2013). The thoracic limb. Small Animal Regional Anesthesia and Analgesia.

[11] Brummett M.C.M., Hong B.E.K., Janda A., Amodeo B.F.S., Lydic R. (2011). Perineural Dexmedetomidine Added to Ropivacaine for Sciatic Nerve Block in Rats Prolongs the Duration of Analgesia by Blocking the Hyperpolarization-activated Cation Current. Anesthesiology.

[12] Brummett M.C.M., Padda B.A.K., Amodeo B.F.S., Welch M.K.B., Lydic R. (2009). Perineural Dexmedetomidine Added to Ropivacaine Causes a Dose-dependent Increase in the Duration of Thermal Antinociception in Sciatic Nerve Block in Rat. Anesthesiology.

[13] Esmaoglu A., Yegenoglu F., Akin A., Turk C.Y. (2010). Dexmedetomidine Added to Levobupivacaine Prolongs Axillary Brachial Plexus Block. Anesth. Analg..

[14] Sarotti D., Rabozzi R., Franci P. (2019). Effects of intravenous dexmedetomidine infusion on local anaesthetic block: A spinal anaesthesia clinical model in dogs undergoing hind limb surgery. Res. Vet. Sci..

[15] Tisotti T., Valverde A., Hopkins A., O’Sullivan M.L., Hanna B., Arroyo L. (2020). Use of intravenous lidocaine to treat dexmedetomidine-induced bradycardia in sedated and anesthetized dogs. Vet. Anaesth. Analg..

[16] Weerink M., Struys M.M.R.F., Hannivoort L.N., Barends C.R.M., Absalom A.R., Colin P. (2017). Clinical Pharmacokinetics and Pharmacodynamics of Dexmedetomidine. Clin. Pharmacokinet..

[17] Kuusela E., Raekallio M., Anttila M., Falck I., Mölsä S., Vainio O.M. (2000). Clinical effects and pharmacokinetics of medetomidine and its enantiomers in dogs. J. Vet. Pharmacol. Ther..

[18] Dent B.T., Aarnes T.K., Wavreille V., Lakritz J., Lerche P., KuKanich B., Pereira C.H.R., Bednarski R.M. (2019). Pharmacokinetics and pharmacodynamic effects of oral transmucosal and intravenous administration of dexmedetomidine in dogs. Am. J. Vet. Res..

[19] Viscasillas J., Seymour C.J., Brodbelt D.C. (2013). A cadaver study comparing two approaches for performing maxillary nerve block in dogs. Vet. Anaesth. Analg..

[20] Toutain P.-L., Bousquet-Melou A. (2004). Plasma terminal half-life. J. Vet. Pharmacol. Ther..

[21] European Medicines Agency European Public Assessment Report. https://www.ema.europa.eu/en/documents/product-information/dexdomitor-epar-product-information_en.pdf.

[22] Di Cesare F., Gioeni D., Ravasio G., Pellegrini A., Lucatello L., Bisutti V., Villa R., Cagnardi P. (2019). Clinical pharmacokinetics of a dexmedetomidine–methadone combination in dogs undergoing routine anaesthesia after buccal or intramuscular administration. J. Vet. Pharmacol. Ther..

[23] Dutta S., Lal R., Karol M.D., Cohen T., Ebert T. (2000). Influence of Cardiac Output on Dexmedetomidine Pharmacokinetics. J. Pharm. Sci..

[24] Avram M.J., Krejcie T.C., Niemann C.U., Enders-Klein C., Shanks C.A., Henthorn T.K. (2000). Isoflurane Alters the Recirculatory Pharmacokinetics of Physiologic Markers. Anesthesiology.

[25] Di Bella C., Skouropoulou D., Stabile M., Muresan C., Grasso S., Lacitignola L., Valentini L., Crovace A., Staffieri F. (2020). Respiratory and hemodynamic effects of 2 protocols of low-dose infusion of dexmedetomidine in dogs under isoflurane anesthesia. Can. J. Vet. Res..

[26] Kersten J., Pagel P.S., Tessmer J.P., Roerig D.L., Schmeling W.T., Waritier D.L. (1993). Dexmedetomidine Alters the Hemodynamic Effects of Desflurane and Isoflurane in Chronically Instrumented Dogs. Anesthesiology.

[27] Grasso S.C., Ko J.C., Weil A.B., Paranjape V., Constable P.D. (2015). Hemodynamic influence of acepromazine or dexmedetomidine premedication in isoflurane-anesthetized dogs. J. Am. Vet. Med. Assoc..

[28] Akashi N., Murahata Y., Hosokawa M., Hikasa Y., Okamoto Y., Imagawa T. (2021). Cardiovascular and renal effects of constant rate infusions of remifentanil, dexmedetomidine and their combination in dogs anesthetized with sevoflurane. J. Vet. Med. Sci..

[29] Kuusela E., Raekallio M., Hietanen H., Huttula J., Vainio O. (2002). 24-hour Holter-Monitoring in the Perianaesthetic Period in Dogs Premedicated with Dexmedetomidine. Vet. J..

[30] Congdon J.M., Marquez M., Niyom S., Boscan P. (2011). Evaluation of the sedative and cardiovascular effects of intramuscular administration of dexmedetomidine with and without concurrent atropine administration in dogs. J. Am. Vet. Med. Assoc..

[31] Murrell J.C., Hellebrekers L.J. (2005). Medetomidine and dexmedetomidine: A review of cardiovascular effects and antinociceptive properties in the dog. Vet. Anaesth. Analg..

[32] Pypendop B.H., Verstegen J.P. (1998). Hemodynamic effects of medetomidine in the dog: A dose titration study. Vet. Surg..

[33] Heppolette C.A.A., Brunnen D., Bampoe S., Odor P.M. (2020). Clinical Pharmacokinetics and Pharmacodynamics of Levobupivacaine. Clin. Pharmacokinet..

